# The Evolution of Cognitive Impairment and Wellbeing Research: A Bibliometric Analysis

**DOI:** 10.3390/healthcare14152243

**Published:** 2026-07-23

**Authors:** Shengyuan Xu, Shaojie Qi

**Affiliations:** 1 Graduate School of Information Sciences, Tohoku University, Aoba-ku, Sendai 980-8579, Japan; 2 Research Institute of Social Development, Southwestern University of Finance and Economics, Chengdu 611130, China; qishaojie@swufe.edu.cn

**Keywords:** cognitive impairment, well-being, bibliometric analysis, collaboration network, research hotspots

## Abstract

**Highlights:**

**What are the main findings?**

- This study found that research on cognitive impairment and well-being has developed a dual-core knowledge structure centered on caregiver well-being and the psychosocial well-being of people with cognitive impairment, with non-pharmacological interventions, long-term care contexts, and methodological research serving as key connecting themes.
- Collaboration networks showed a markedly uneven structure, with the United States acting as a scale hub, the United Kingdom functioning as a bridging hub, and China displaying a high-output but relatively low-connectivity pattern.

**What are the implications of the main findings?**

- Research on cognitive impairment is shifting from a disease-oriented perspective toward a well-being-oriented approach, with caregiver support, non-pharmacological interventions, and technology-enabled strategies likely to become major future directions.
- Future research should strengthen economic analyses, global equity perspectives, and evidence generation in low- and middle-income countries to address major gaps in the current knowledge base.

**Abstract:**

**Background/Objectives**: Cognitive impairment and well-being have become increasingly connected in aging research, yet the intellectual structure, collaboration patterns, and thematic evolution of this interdisciplinary field remain insufficiently mapped. This study aimed to provide a bibliometric overview of research on cognitive impairment and well-being. **Methods**: A total of 1355 publications published between 1985 and 2026 were retrieved from the Web of Science Core Collection. Bibliometric analyses were conducted using VOSviewer and CiteSpace to examine publication trends, journal distribution, collaboration networks, disciplinary evolution, keyword clusters, burst keywords, and document co-citation patterns. **Results**: The field showed a clear pattern of accelerated growth, especially during the past decade. The knowledge structure was organized around two core themes: caregiver well-being and the psychosocial well-being of people with cognitive impairment. Non-pharmacological interventions, long-term care contexts, and methodological research formed connecting clusters around these themes. Caregiver burden emerged as the earliest major research line and has recently moved toward evidence-based intervention development. Non-pharmacological interventions showed a three-stage evolution, from single-modality explorations such as music therapy, to meta-analysis-driven evidence synthesis, and then to technology-enabled approaches such as virtual reality. Collaboration network analysis showed that the USA acted as a scale hub, the United Kingdom as a bridging hub, and China as a high-output but low-connectivity participant. Bridging authors, including Orrell and Bennett, occupied structural hole positions. **Conclusions**: This study provides a four-decade knowledge map of cognitive impairment and well-being research and offers a reference framework for theoretical integration and future research planning.

## 1. Introduction

The accelerating aging of the global population has made cognitive impairment and related cognitive disorders one of the most challenging public health issues of the twenty-first century. At the same time, the global economic burden associated with cognitive disorders continues to increase. According to statistics released by Alzheimer’s Disease International (ADI), the annual global societal cost of dementia was estimated at US$1.3 trillion in 2019. Approximately half of this cost was attributable to care provided by informal caregivers, including family members and friends, while the remaining share was largely associated with direct expenditures on healthcare and social care. Without effective responses, the total cost is projected to reach US$2.8 trillion by 2030 [1]. The World Health Organization (WHO) identified dementia as a public health priority and highlighted its substantial impact on mortality, disability, long-term care needs, healthcare systems, and social support networks worldwide [2].

Faced with this challenge, the focus of researchers and policymakers has gradually extended beyond disease treatment toward a broader concern with health maintenance and support. The 2020 Lancet Commission report, *Dementia prevention, intervention, and care: 2020 report of the Lancet Commission*, identified twelve modifiable risk factors and proposed that interventions targeting these factors throughout the life course could theoretically prevent or delay approximately 40% of dementia cases [3]. The report also emphasized that, in the absence of curative treatments, improving the quality of life of people living with dementia and their caregivers, supporting caregivers, and strengthening care systems should be regarded as important goals of dementia intervention and care [3]. Against this background, well-being has evolved from a psychological construct into an important outcome in research on cognitive health in later life. In the present review, well-being is used in a broad sense that encompasses quality of life, mental health, positive affect, and social participation. Within dementia care, patients and caregivers often place considerable value on maintaining quality of life, preserving social relationships, supporting everyday functioning, and receiving appropriate care and support when defining care goals [4]. This perspective reflects a broader shift in cognitive impairment research from a predominantly biomedical orientation toward social, psychological, and ecological perspectives. It also resonates with Ryff’s multidimensional framework of psychological well-being [5], which includes: autonomy, environmental mastery, personal growth, positive relations with others, purpose in life, and self-acceptance. Although the framework was not originally developed for people living with cognitive impairment, it has provided an important theoretical reference for research on psychological well-being in later life. Alongside this shift, the range of disciplines involved in research on cognitive impairment and well-being has continued to expand. Gerontology and geriatrics remain foundational disciplines in this field. Psychiatry, nursing, psychology, public health, neuroscience, social work, and medical informatics have become increasingly engaged in related research. Psychiatric comorbidity may also increase cognitive vulnerability through metabolic pathways linked with cognitive decline and dementia [6]. Neuroimaging evidence on psychiatric disorders further supports a neuropsychiatric view of cognitive impairment and well-being [7]. Together, these developments have produced a research landscape that is highly interdisciplinary while also characterized by considerable thematic fragmentation.

However, when confronted with a large and expanding body of literature, researchers often find it difficult to grasp the field’s overall knowledge structure, the patterns of collaboration networks, and the evolving trajectory of research hotspots within a short period of time. Bibliometric analysis can, to some extent, compensate for the limited macro-level perspective of traditional systematic reviews. As a systematic method grounded in statistical analysis and network-based analysis, bibliometrics can process large-scale scientific literature data and reveal the knowledge structure and developmental trajectory of a given research field through quantitative analyses of publication trends, collaboration networks, keyword co-occurrence, co-citation relationships, and burst detection [8,9]. For this reason, using bibliometric methods to produce a systematic and comprehensive map of this field is both methodologically justified and practically necessary. According to our current search results, existing bibliometric studies have mainly focused on Alzheimer’s disease, dementia care, psychological well-being, or related intervention topics. A systematic study that uses the Web of Science Core Collection as its data source, treats the intersection between “cognitive impairment” and “well-being” as the core unit of analysis, and traces its knowledge evolution and collaboration structure from 1985 to 2026 remains limited. Against this background, the present study uses CiteSpace and VOSviewer as complementary bibliometric tools to conduct a macro-level analysis across an extended time span from 1985 to 2026 and across multiple analytical dimensions. In doing so, it addresses gaps in existing research concerning database coverage, conceptual integration, and temporal depth [10].

Accordingly, this study aims to conduct a systematic macro-level analysis of the intersection between cognitive impairment and well-being through bibliometric methods. The study seeks to: (1) trace growth trends in annual publication output and the staged evolution of disciplinary structure; (2) examine collaboration networks at the levels of countries, institutions, and authors, together with the patterns of knowledge production reflected in these networks; (3) identify major keyword clusters and the evolutionary pathways of research hotspots; and (4) detect research frontiers and potential future trends through burst detection and co-citation analysis.

## 2. Materials and Methods

### 2.1. Data Source

Following recommendations to specify Web of Science Core Collection (WoSCC) datasets and their coverage timespans [11], this study used three citation indexes: the Science Citation Index Expanded (1900 to present), the Social Sciences Citation Index (1900 to present), and the Arts and Humanities Citation Index (1975 to present). Literature retrieval and data download were conducted on 15 April 2026. WoSCC was selected because it provides standardized bibliographic records and cited-reference data that are widely used in bibliometric analysis and science mapping [8,9]. Its standardized bibliographic format is also highly compatible with CiteSpace and VOSviewer, which are commonly used to visualize co-citation, collaboration, and co-occurrence networks [12,13]. The rigorous journal selection mechanism of WoSCC ensures a basic quality threshold for the literature included in the database, which is particularly important for scientometric research that relies on citation network analysis [8]. Previous studies have also shown that substantial differences exist in journal coverage across major citation databases, making database selection an important methodological consideration in bibliometric analysis [10].

### 2.2. Search Strategy

The search strategy adopted a combined search using the TS field and the TI field. The search query was as follows: *TS = (“well-being” OR “wellbeing” OR “life satisfaction” OR “happiness”) AND TI = (“dementia” OR “alzheimer” OR “cognitive impair” OR “cognitive decline” OR “MCI” OR “memory loss”)*. No restriction was imposed on the publication period. Document types were limited to Article and Review Article, and the language was restricted to English. The placement of well-being-related terms in the TS field (including titles, abstracts, author keywords, and Keywords Plus) for broad retrieval was based on a methodological reality of this area of research: well-being often appears as a research variable or secondary outcome in abstracts and keywords, and restricting these terms to the title field would lead to the systematic omission of a substantial number of relevant empirical studies. At the same time, topic-field retrieval in WoSCC may be affected by the limited availability of abstracts and keyword fields in older records, so early-stage publication patterns need to be interpreted with caution [14]. The placement of cognitive impairment-related terms in the TI field (titles only) for precise identification was intended to ensure that the included literature treated cognitive impairment as the primary research focus. If cognitive impairment-related terms were also placed in the TS field, a large number of studies in which cognitive impairment appeared only as a covariate or research background would be retrieved. This would introduce substantial noise into the dataset and weaken the semantic focus of subsequent co-citation analysis and keyword clustering. This asymmetric field design improved thematic specificity, but it may also have made studies centered on well-being, with cognitive decline appearing only as a covariate or contextual factor, less visible in the final dataset.

### 2.3. Literature Screening

A manual screening process was conducted for the 3560 initially retrieved records according to a systematic literature screening procedure, as shown in Figure S1. The exclusion criteria included non-original research publications such as conference abstracts, editorial materials, book reviews, news items, and corrections; publications that were confirmed, after title and abstract screening, to have no substantive relevance to cognitive impairment or well-being; and studies primarily based on animal experiments or in vitro experiments. After screening, 1367 records were retained. These records were imported into EndNote for automatic duplicate removal to identify possible repeated records caused by Early Access/final publication versions or minor metadata differences, followed by manual record-by-record verification to ensure that no duplicate records remained. A final set of 1355 publications was identified as the analytical dataset. The initial screening was conducted by the first author, and the final included records were checked and confirmed by the second author. The data download format was set as “full record and cited references” to ensure the completeness of citation data required for subsequent co-citation analysis.

### 2.4. Analytical Methods and Strategy

This study used VOSviewer (version 1.6.20) and CiteSpace (version 6.4.R1) in combination to conduct multidimensional knowledge mapping. These tools have been widely used in bibliometric and scientometric studies to visualize intellectual structures, collaboration networks, co-citation patterns, and thematic evolution [12,13]. The two tools complemented each other in function: the distance-based visualization algorithm of VOSviewer is suitable for presenting the clustering structure and density distribution of large-scale networks, while the built-in burst detection and time-zone evolution analysis functions of CiteSpace are particularly suitable for tracing the dynamic shifts in research frontiers. The selection of analytical indicators and the setting of parameters followed the methodological consensus at the frontier of this field. In CiteSpace, the time slice was set to one year, the node threshold was selected using the g-index, and network pruning was conducted using the Pathfinder algorithm to highlight the backbone structure of the network. In the keyword co-occurrence analysis conducted with VOSviewer, the minimum occurrence threshold was set to 5.

## 3. Results

### 3.1. Publication Output, Publishing Journals, and Collaboration Networks

**Figure 1:**
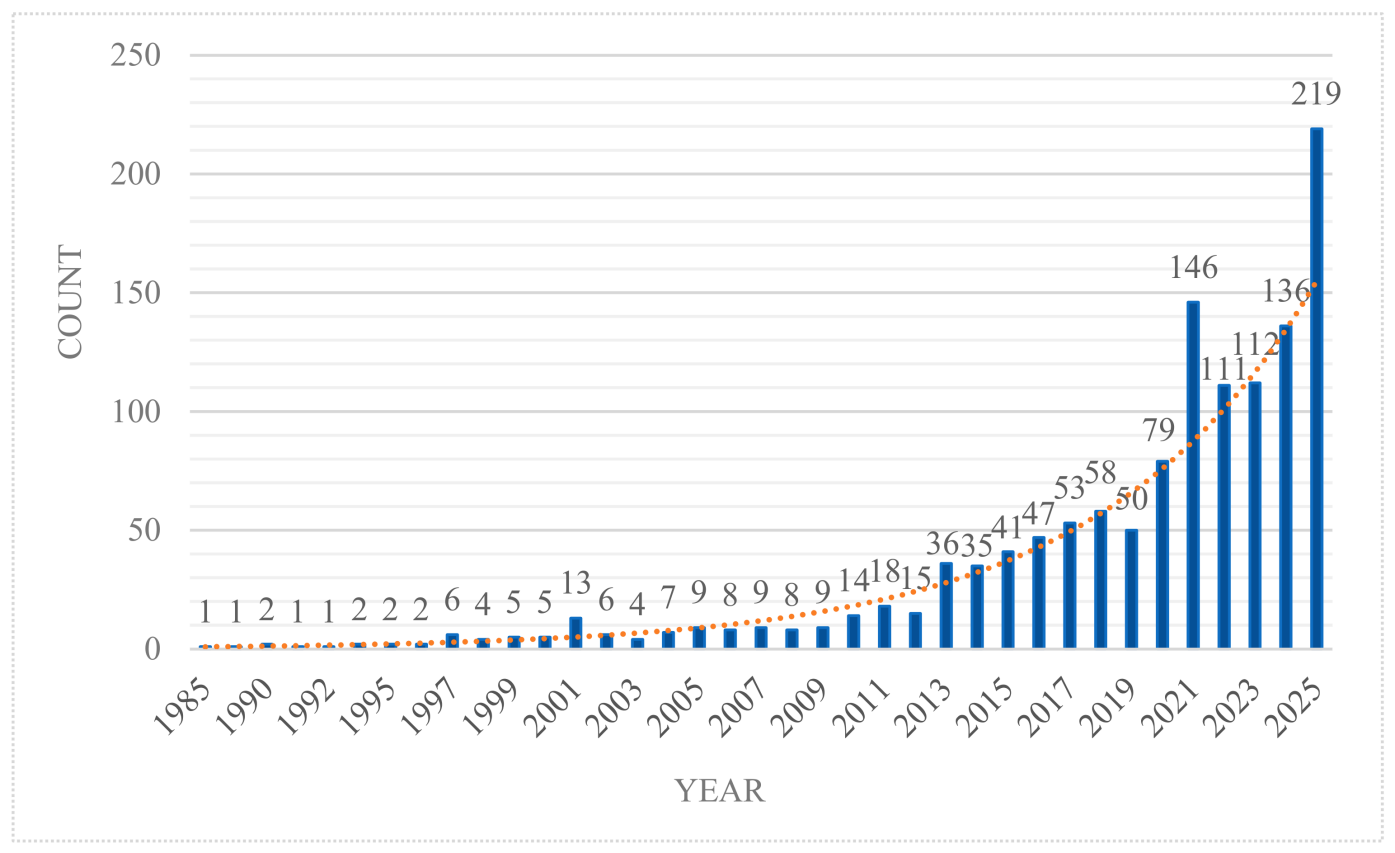
Annual Publication Trends.

Figure 1 shows the changes in annual publication output between 1985 and 2025. The earliest relevant study can be traced back to 1985, after which the number of publications remained at a relatively low level for an extended period. During the 1980s and 1990s, annual publication output generally fluctuated within single digits (This low early output should be interpreted with caution because earlier WoSCC records may have less complete abstract, keyword, and author-address information). After the beginning of the twenty-first century, scholarly attention to this topic gradually increased, accompanied by a slow rise in publication output. The annual number of publications reached 13 in 2001 and remained at approximately 10 publications per year between 2005 and 2012. After 2013, the growth trend became more pronounced, with annual publication output increasing from 15 publications in 2012 to 36 publications in 2013. This turning point broadly coincided with the timing of the *G8 Dementia Summit*, which formally proposed a global initiative to combat dementia in 2013 [15], suggesting that major international policy agendas may have influenced the allocation of research funding and the distribution of scholarly attention within this field. The annual publication output reached 79 in 2020, after which the scale of research expanded further. Annual publication output exceeded 100 publications for the first time, reaching 219 publications in 2025. The annual publication output increased sharply to 146 records in 2021, a peak that should be interpreted descriptively and may partly reflect the concentration of pandemic-related dementia-care studies on well-being, social isolation, service disruption, and technology-supported care [1,16,17]. Annual publication output showed a clear upward trend during the observed period, with particularly rapid growth in the past decade. Overall, the development of this field does not represent a simple linear accumulation of knowledge. Instead, it exhibits characteristics of event-driven exponential growth, with research activity increasing substantially during the past decade alongside the advancement of global policy agendas related to population aging.

Research outputs in this field were published in 200 different journals. The top 15 journals by publication output are shown in Table 1. *Dementia: International Journal of Social Research and Practice* (76 publications), *Aging & Mental Health* (48 publications), and *BMC Geriatrics* (47 publications) were the three journals that published the largest number of papers on this topic. *Journal of Alzheimer’s Disease* (39 publications), *The Gerontologist* (35 publications), and *International Journal of Geriatric Psychiatry* (34 publications) ranked fourth to sixth, respectively. *International Psychogeriatrics* (30 publications) ranked seventh, while *Journal of Clinical Nursing* (23 publications) ranked eighth. *Frontiers in Psychiatry*, *International Journal of Environmental Research and Public Health*, *Journals of Gerontology, Series B: Psychological Sciences and Social Sciences*, and *PLOS ONE* each published 21 papers and were tied for ninth place. *Frontiers in Psychology*, *Geriatric Nursing*, and *Journal of Advanced Nursing* each published 17 papers and were also among the leading journals. In terms of journal distribution, research outputs in this field were highly concentrated in specialized journals related to gerontology, geriatric psychiatry, nursing, and public health, indicating that its publication venues span multiple domains, including biomedicine, psychosocial research, and health services, with clear interdisciplinary characteristics.

### 3.2. Collaboration Networks and Performance Analysis

**Figure 2:**
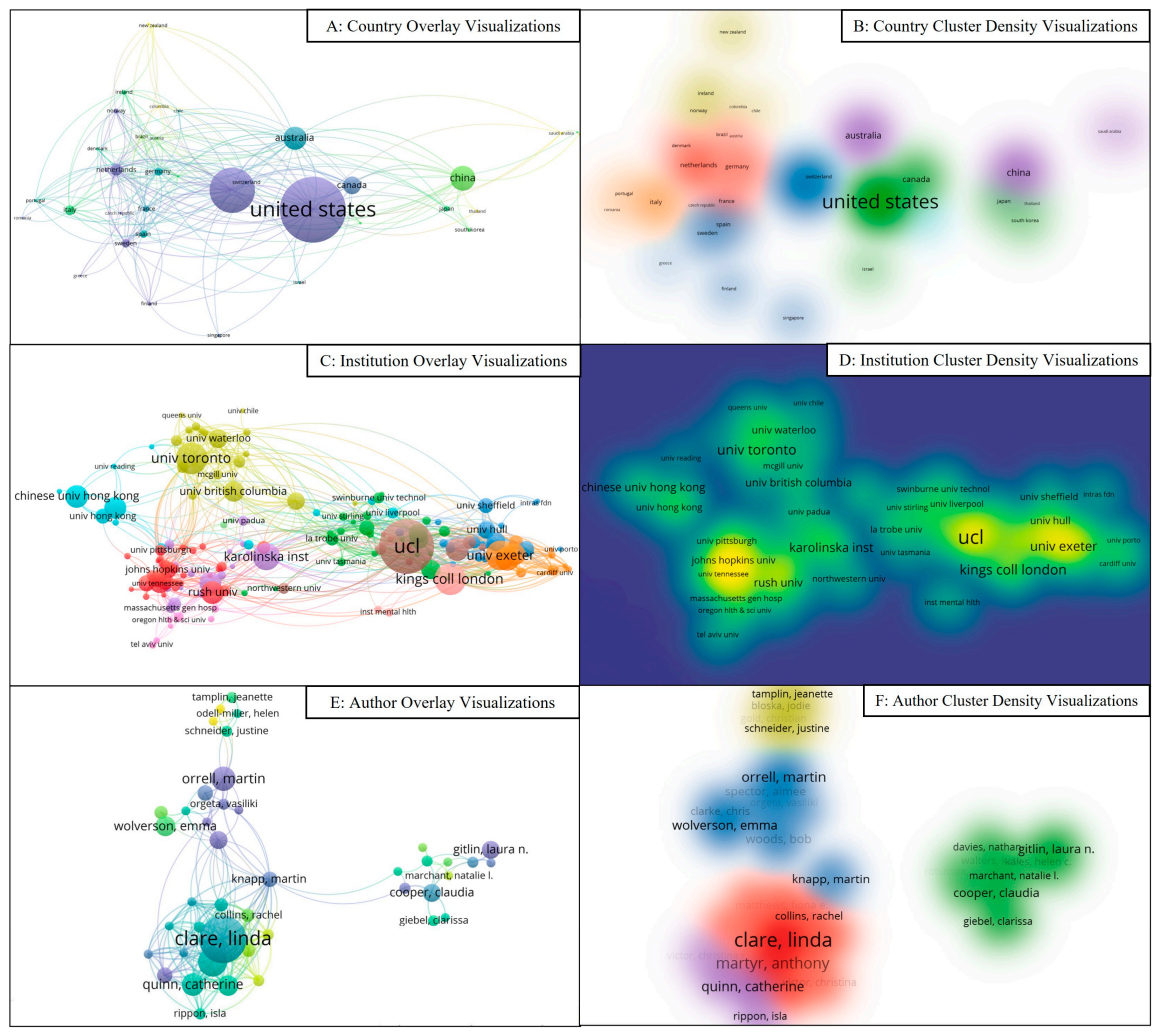
Collaboration Networks and Density Maps of Countries, Institutions, and Authors.

The distribution of researchers, their affiliated institutions, and countries can partly reflect the pattern of knowledge production and the collaboration structure in this field. The top 10 countries (out of 80), institutions (out of 1364), and authors (out of 6549) by publication output are shown in Table 2. At the country level, the USA ranked first with 403 publications and a betweenness centrality (BC) of 0.32. The United Kingdom ranked second with 244 publications (BC = 0.34), followed by Australia (142 publications, BC = 0.30), China (132 publications, BC = 0.10), and Canada (111 publications, BC = 0.05). The United Kingdom had a higher betweenness centrality than the USA, suggesting a relatively more prominent bridging position in connecting research networks across Europe, Commonwealth countries, and North America. This may be related to its dual position of participating in research collaborations under European Union frameworks while maintaining long-standing close academic ties with North America. North American and European countries remained dominant in this network (Figure 2).

At the institutional level, the University of London had the highest publication output (77 publications, BC = 0.11), followed by University College London (51 publications) and the University of California System (49 publications). The University of California System had the highest betweenness centrality (BC = 0.14). The University of New South Wales Sydney and Vrije Universiteit Amsterdam both had a centrality value of 0.08, also showing a certain degree of network connectivity (Figure 2). At the author level, Clare, L. ranked first with 21 publications and had a betweenness centrality of 0.27, combining publication scale with network connectivity. Bennett, D. A. and Martyr, A. each published 13 papers, with Bennett’s betweenness centrality reaching 0.47, indicating that he played a role in both output and connectivity. Orrell, M. published 12 papers and had a betweenness centrality as high as 0.79, making him the most prominent node in the network in terms of connectivity. His high connectivity suggests that he occupied an important bridging position within the co-authorship network. Boyle, P. A. and Quinn, C. each published 10 papers. Subramaniam, M., Abdin, E., Vaingankar, J. A., and Wolverson, E. each published 9 papers. A certain degree of differentiation was observed within the group of highly productive authors. Authors such as Martyr, A. (BC = 0) and Subramaniam, M. (BC = 0) had zero centrality, indicating that their work was mainly embedded in existing collaboration circles or relatively independent teams. Orrell and Bennett, by contrast, combined high productivity with high connectivity. This difference suggests that although a group of core authors has emerged in this field, the overall collaboration network remains relatively loose, and a stable collaborative community has not yet fully formed.

### 3.3. Disciplinary Development and Interdisciplinary Characteristics

**Figure 3:**
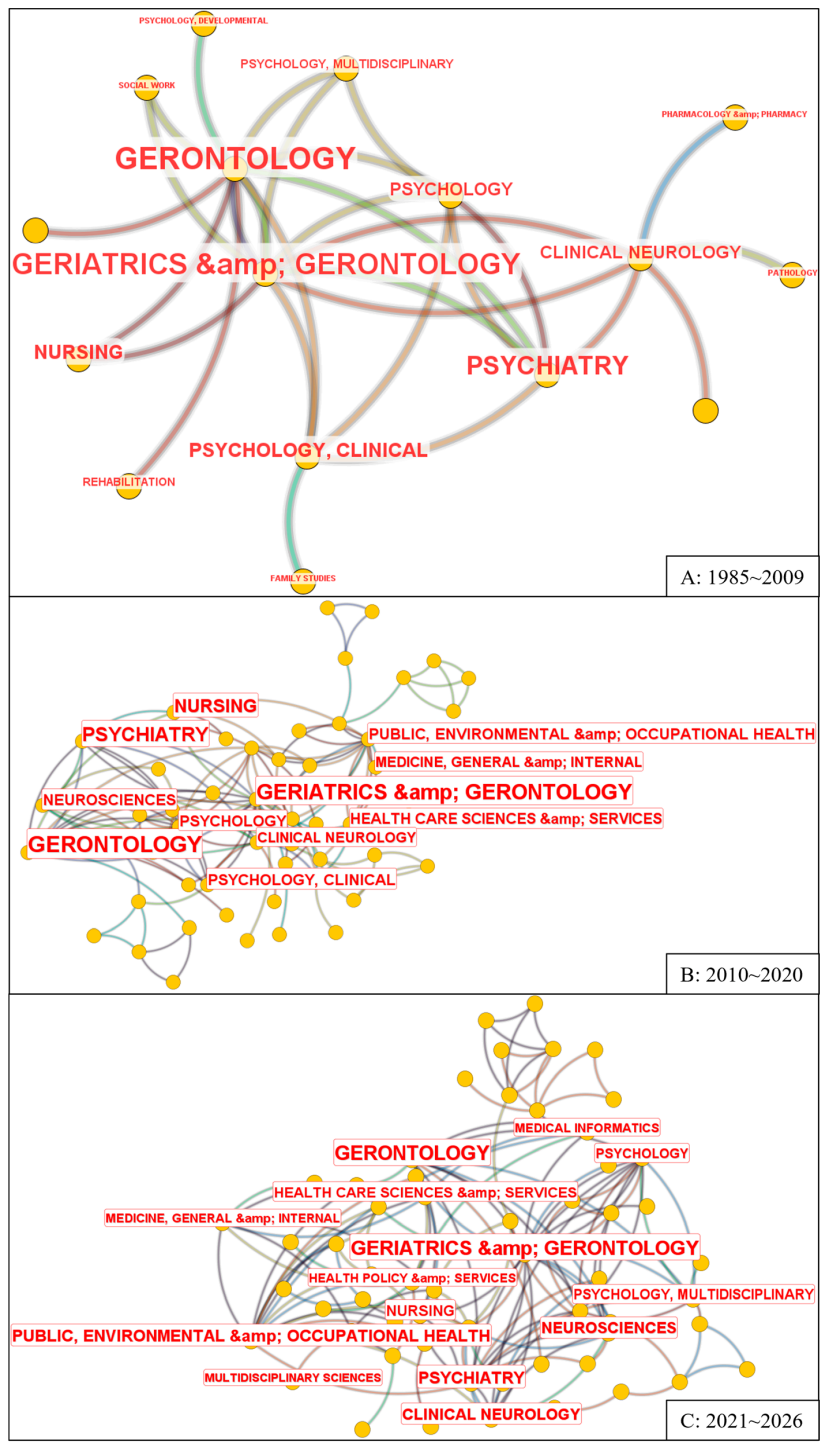
Disciplinary Collaboration Network. Notes: This figure was generated using CiteSpace 6.4.R1. In some Web of Science disciplinary labels, the ampersand symbol “&” may be displayed as the HTML entity “&amp;” in the software output; it should be read as “&”.

Figure 3 shows the staged evolution of disciplinary distribution in this field. The publication frequency and betweenness centrality of disciplinary categories at each stage are shown in Table 3. The period from 1985 to 2009 can be regarded as the disciplinary emergence phase. During this stage, research was mainly grounded in *GERONTOLOGY* and *GERIATRICS and GERONTOLOGY*. These two disciplines were prominent in both publication output and betweenness centrality, forming the intellectual backbone of the field at that time. *CLINICAL NEUROLOGY* entered the network in 1990 and showed a certain degree of betweenness centrality. Disciplines such as FAMILY STUDIES appeared only sporadically. Overall, interdisciplinary integration remained highly limited, and the disciplinary structure was still characterized by a monocentric emergence pattern centered on gerontology.

The period from 2010 to 2020 was the development phase, during which annual publication output increased steadily from 14 to 79 publications. *GERONTOLOGY* and *GERIATRICS and GERONTOLOGY* continued to occupy dominant positions, ranking near the top in both publication output and centrality. The publication output of PSYCHIATRY rose to third place, while the participation of *NURSING*, *PSYCHOLOGY*, and *PSYCHOLOGY, CLINICAL* also increased markedly. It is worth noting that *PUBLIC, ENVIRONMENTAL and OCCUPATIONAL HEALTH*, although ranking only seventh in publication output, reached a betweenness centrality of 0.41, making it the most important interdisciplinary bridging discipline during this stage. This indicates that the field underwent a shift from a disease-centered model toward a biopsychosocial model that incorporated social and environmental factors. Public health effectively connected gerontology, psychiatry, nursing, and other directions into a more integrated knowledge network. At the same time, *NEUROSCIENCES*, *HEALTH POLICY and SERVICES*, and *MEDICINE, GENERAL and INTERNAL* entered the field successively, and the research perspective expanded from a gerontology-centered core toward peripheral health sciences. Interdisciplinary integration began to take shape.

The period from 2021 to 2026 was the rapid expansion phase. The participation of *PSYCHIATRY*, *PUBLIC, ENVIRONMENTAL and OCCUPATIONAL HEALTH*, *NEUROSCIENCES*, *PSYCHOLOGY*, *MULTIDISCIPLINARY*, and *SOCIAL WORK* increased substantially. *MEDICAL INFORMATICS* entered the network for the first time and immediately reached 29 publications, with a betweenness centrality of 0.21, becoming an emerging interdisciplinary node. Development during this period was driven by both technological and social factors. Computational sciences represented by medical informatics, together with social sciences represented by social work and psychology, became embedded at multiple points within the traditional gerontology system. This promoted the evolution of the field toward the integration of precision assessment and human-centered care. At this point, a pattern of deep multidisciplinary integration had formed, with *GERONTOLOGY* and *GERIATRICS and GERONTOLOGY* as the foundation, neuropsychiatric health and public health as two major supporting directions, and medical informatics and social sciences as emerging growth points.

### 3.4. Top 10 Cited Journals

The citation frequency of a literature source or journal is often regarded as an important indicator of its academic influence within a specific research field [9]. Citation analysis can reveal knowledge diffusion relationships among different publications and help identify important knowledge sources within a field. With tools such as VOSviewer, the citation relationships among journals can be presented in network form, thereby helping locate core journals and their academic connections. In terms of citation frequency, the core knowledge sources in this field showed clear interdisciplinary characteristics. The results of the journal citation analysis are shown in Table 4. The most frequently cited journal was *The Gerontologist* (3151 citations), followed by *Cochrane Database of Systematic Reviews* (2481 citations) and *Dementia: International Journal of Social Research and Practice* (1536 citations). These highly cited journals were mainly concentrated in the fields of gerontology, evidence-based medicine, and social research on dementia, indicating that aging-related health, intervention evidence synthesis, and care practice constitute important knowledge foundations of this field.

At the same time, several journals focusing on mental health and nursing also showed strong influence, such as *Aging & Mental Health* (1508 citations), *International Psychogeriatrics* (1345 citations), and *American Journal of Geriatric Psychiatry* (1171 citations). In addition, clinical and nursing journals also occupied important positions, including *International Journal of Geriatric Psychiatry* (1135 citations), *Journal of Clinical Nursing* (1081 citations), and *Journal of the American Geriatrics Society* (1029 citations), reflecting the importance of psychiatric care and clinical nursing research within the knowledge system of this field. Overall, highly cited journals in this field were mainly distributed across gerontology, psychiatry, nursing, and evidence-based medicine, forming a stable interdisciplinary knowledge source structure centered on cognitive and mental health in later life.

### 3.5. Keyword Clustering Timeline View and Burst Detection

**Figure 4:**
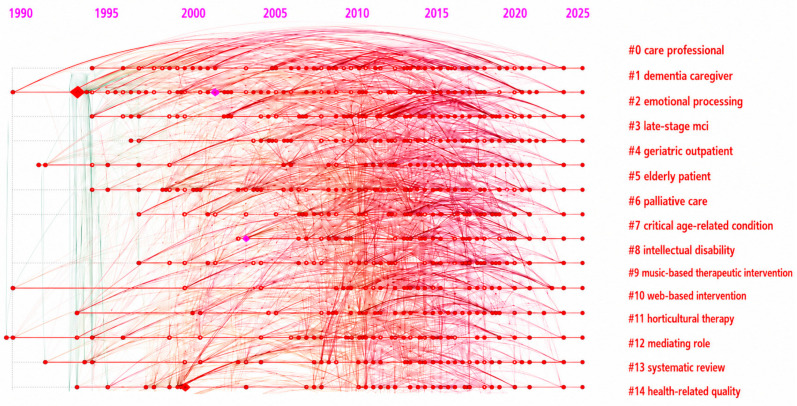
Timeline Visualization of Keyword Clusters. Notes: The colors of the nodes and links indicate temporal progression from earlier years in green to more recent years in red. Purple rings indicate nodes with high betweenness centrality, while bright red rings indicate keywords with occurrence bursts.

To further reveal the knowledge structure and research frontiers in the field of cognitive impairment and well-being, this study conducted a keyword clustering analysis. The top 20 keywords by occurrence frequency are shown in Table 5, and Figure 4 presents several core knowledge clusters and their temporal distribution in this field. Based on the clustering results, four major research directions can be identified. The first direction concerns caregiver burden and support. This direction is mainly composed of two clusters, #0 *care professional* and #1 *dementia caregiver*, with research attention concentrated on caregivers. The high-frequency keywords “family caregivers” (centrality = 0.40) and “burden” (centrality = 0.57) serve as core markers of this direction. This indicates that the physical, psychological, and social burdens borne by family caregivers have become an independent research object that cannot be ignored within the field, moving to some extent beyond an analytical perspective centered only on patients. The second direction concerns disease pathology and population epidemiology. This direction integrates several clusters, including #3 *late-stage MCI*, #5 *elderly patient*, #7 *critical age-related condition*, and #8 *intellectual disability*, and focuses on the pathogenesis, clinical staging, and population distribution characteristics of cognitive impairment. The high-frequency terms “Alzheimer’s disease” (409 occurrences), “mild cognitive impairment” (160 occurrences), and “cognitive impairment” (165 occurrences, centrality = 0.30) constitute the basic terminology system of this direction. This also represents the most classical knowledge foundation of the field.

The third direction concerns non-pharmacological interventions and health promotion. This direction consists of clusters such as #9 *music-based therapeutic intervention*, #10 *web-based intervention*, #11 *horticultural therapy*, #12 *mediating role*, and #13 *systematic review*. The high-frequency keyword “intervention” (88 occurrences, centrality = 0.24) serves as their shared marker. The expansion of this direction indicates that research in the field is shifting from the description and explanation of cognitive impairment toward active intervention and improvement. Intervention forms have also evolved from single approaches to diversified approaches, and from offline delivery to combined online and offline models, while showing strong attention to evidence-based evaluation. A recent meta-analysis on the effects of mindfulness-based interventions on neuropsychiatric symptoms and psychological well-being in people with subjective cognitive decline and mild cognitive impairment [18] reflects this trend, showing that the field is moving from the exploration of individual interventions toward systematic evidence synthesis. The fourth direction concerns lived experience and healthcare services. This direction covers clusters such as #4 *geriatric outpatient*, #6 *palliative care*, #14 *health-related quality*, and #2 *emotional processing*. The high-frequency terms “quality of life” (256 occurrences), “depression” (162 occurrences, centrality = 0.16), and “care” (157 occurrences, centrality = 0.24) are its core concerns, and the prominence of “quality of life” also supports the placement of *#14 health-related quality* within this direction. This direction represents a humanistic turn in the field, focusing not only on how diseases are treated, but also on patients’ overall experiences, subjective feelings, and quality of life within healthcare systems, as well as the mechanisms through which emotional processing may operate.

**Figure 5:**
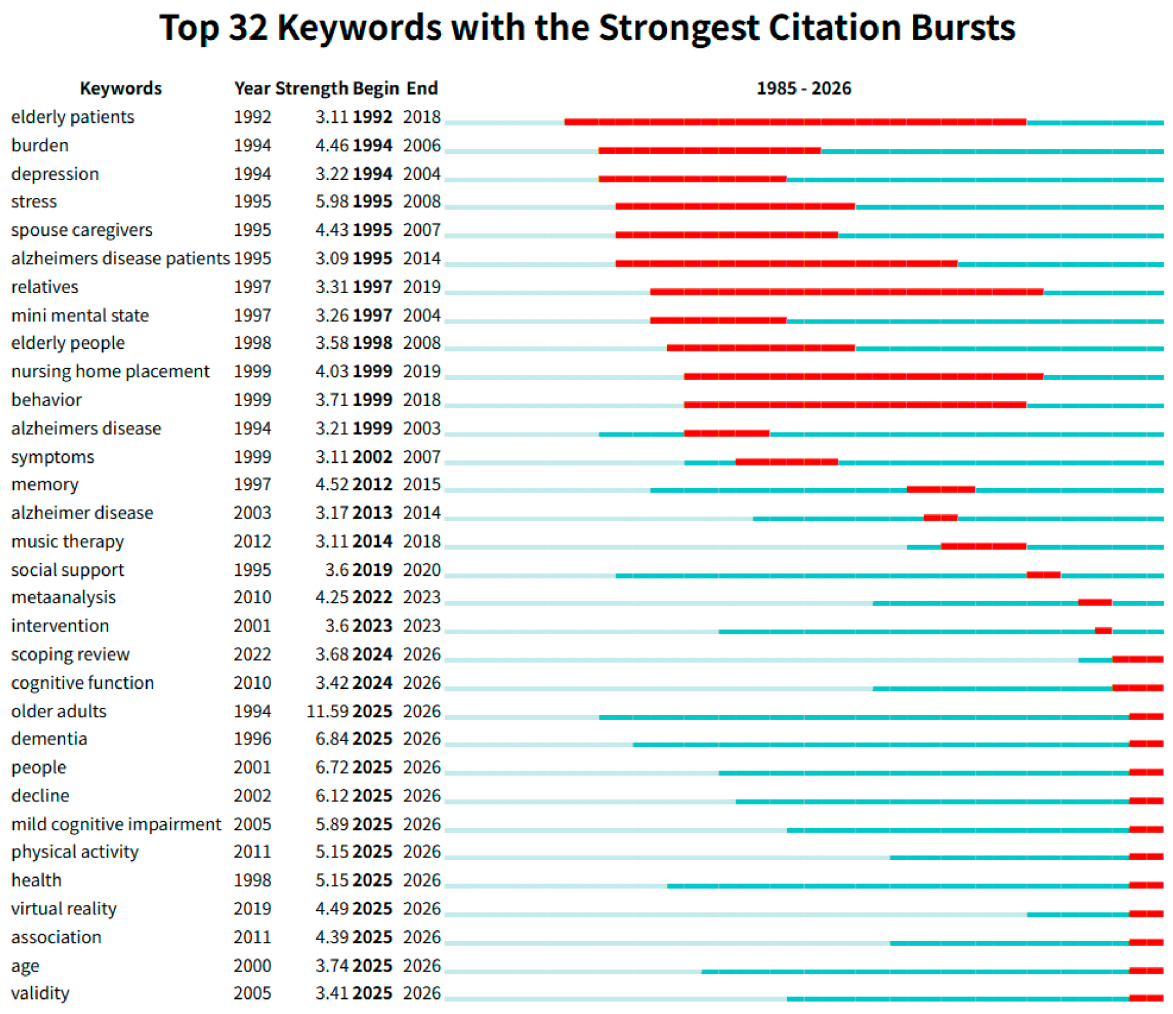
Top Keywords with the Strongest Citation Bursts. Notes: Light blue indicates the period before the first appearance of each keyword, dark blue indicates the non-burst period after its first appearance, and red indicates the detected burst period.

Burst keyword analysis further revealed the dynamic transition of research hotspots, and its staged changes broadly corresponded to the emergence sequence of the four research directions described above. Figure 5 presents the burst keyword analysis. Long-duration bursts of broad population labels were interpreted as sustained terminology rather than short-term topical surges. Early burst keywords were mainly related to caregiving burden and emotional problems, such as “burden” (strength = 4.46, 1994–2006), “stress” (strength = 5.98, 1995–2008), “spouse caregivers” (strength = 4.43, 1995–2007), and “depression” (strength = 3.22, 1994–2004), corresponding to the early rise in research on caregiver burden. Mid-stage burst keywords shifted toward cognitive function and intervention exploration. “memory” (strength = 4.52, 2012–2015), “music therapy” (strength = 3.11, 2014–2018), and “social support” (strength = 3.60, 2019–2020) appeared successively. “meta-analysis” (strength = 4.25, 2022–2023) and “intervention” (strength = 3.60, 2023–2023) further indicate that intervention research gradually moved toward systematic evidence-based evaluation. In the past two years, “scoping review” (strength = 3.68, 2024–2026), “cognitive function” (strength = 3.42, 2024–2026), and “virtual reality” (strength = 4.49, 2025–2026) have become new burst points. Of particular note, core keywords such as “older adults” (strength = 11.59), “dementia” (strength = 6.84), “people” (strength = 6.72), “decline” (strength = 6.12), “mild cognitive impairment” (strength = 5.89), “physical activity” (strength = 5.15), and “health” (strength = 5.15) showed concentrated and continuous bursts during 2025–2026, with all burst strengths exceeding 5.0, forming a dense cluster of frontier signals. This indicates that the current field is moving toward the deep integration of non-pharmacological intervention research and lived-experience-oriented care. A frontier direction is emerging that is supported by digital technology, driven by evidence-based research, and centered on maintaining overall function and improving quality of life. The research focus has gradually shifted from the traditional “disability–intervention” framework toward the maintenance of cognitive well-being across the life course.

### 3.6. Document Co-Citation Analysis

**Figure 6:**
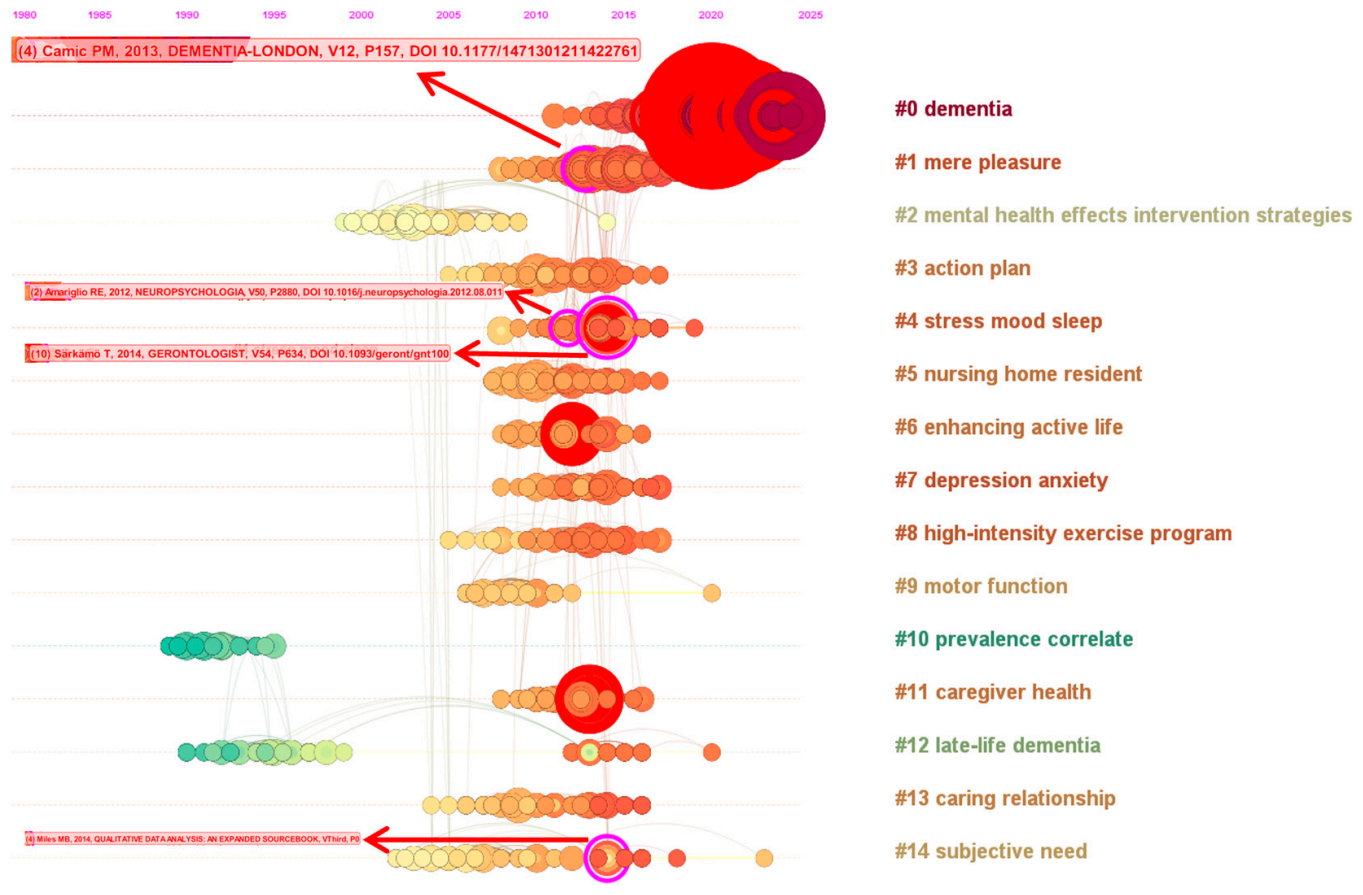
Timeline Visualization of Document Co-Citation Clusters [22,23,26,28].

Document co-citation analysis was used to identify the knowledge base of the field. This study identified 15 clusters, and the representative documents for all 15 clusters are provided in Table S1. These knowledge communities can be integrated along a progressive knowledge chain (Figure 6). The first layer concerns epidemiological evidence and the confirmation of disease burden. Livingston et al. (*The Lancet*, 2020, 74 citations) [3], which focused on dementia prevention, intervention, and care, and Nichols et al. (*The Lancet Public Health*, 2022, 44 citations) [19], which focused on the estimation and forecasting of global dementia prevalence, constituted the highly cited core. The Alzheimer’s Association annual reports (2020–2023) [20] and the WHO report *Dementia: A Public Health Priority* (2012) [21] also occupied important positions. Clusters #0 *dementia*, #10 *prevalence correlate*, and #12 *late-life dementia* all centered on dementia epidemiology and risk factors. Together, these documents established the basic consensus that cognitive impairment is preventable and that risk factors operate across the life course, providing a starting point for subsequent causal inference and intervention design. The second layer concerns mechanism understanding and the refinement of intervention targets. Clusters #4 *stress mood sleep* and #7 *depression anxiety* focused on the intersection between emotion, sleep, and cognitive health. The studies by Amariglio et al. (*Neuropsychologia*, 2012) [22] and Särkämö et al. (*The Gerontologist*, 2014) [23] showed relatively high betweenness centrality. They respectively connected research on subjective cognitive complaints and music-based intervention research, linking subjective experience, emotional states, sleep-related indicators, and objective cognitive decline processes in a more systematic way, and providing operable potential targets for non-pharmacological interventions.

The third layer concerns intervention strategy development and systematic evaluation. Clusters #2 *mental health effects intervention strategies*, #8 *high-intensity exercise program*, and #9 *motor function* focused on the effects of exercise and psychosocial interventions on the maintenance of cognitive function. Woods et al. (*Cochrane Database of Systematic Reviews*, 2018, 13 citations, centrality = 0.10) [24] conducted a systematic review of reminiscence therapy for dementia and became an important node in the intervention evidence chain. The fourth layer concerns real-world care practice and institutional and methodological responses. Clusters #5 *nursing home resident* and #11 *caregiver health* focused on institutional care and caregiver health. Giebel et al. (*International Journal of Geriatric Psychiatry*, 2021, 14 citations) [25] examined the impact of social support service closures on the mental health of dementia caregivers during the COVID-19 pandemic. In essence, this work tested existing intervention evidence in real-world care contexts and responded to the practical issue of translating knowledge into practice. Clusters #3 *action plan*, #6 *enhancing active life*, and #13 *caring relationship* constructed an institutional research network from the perspectives of policy action, active aging, and care relationships. In cluster #14 *subjective need*, Miles, Huberman, and Saldaña’s *Qualitative Data Analysis: A Methods Sourcebook* (3rd ed.) (2014) [26] appeared as a classic methodological work with a purple-ring marker, indicating that qualitative research methods have played a sustained bridging role in this field and have supplemented quantitatively dominated research with a perspective for understanding individual subjective experience. In cluster #1 *mere pleasure*, Camic et al. (*Aging and Mental Health*, 2014) [27] focused on the experiences of viewing and making art, as well as feelings of pleasure, among people with dementia in an art gallery setting. This work opened up a distinctive research direction that moves beyond functional improvement and attaches importance to the quality of lived experience and subjective pleasure.

## 4. Discussion

### 4.1. Interpreting Publication Growth, Journal Distribution, and Collaboration Patterns

At the national level, the USA and the United Kingdom have long occupied dominant positions within the collaboration network, although their roles are clearly differentiated. The USA ranked first with 403 publications. Its position within the collaboration network can be interpreted as closer to that of a “scale hub”, with its central position supported by substantial research investment and clusters of highly productive institutions. The United Kingdom ranked second with 244 publications, yet its betweenness centrality (0.34) was slightly higher than that of the USA (0.32). This suggests that the United Kingdom may play more of a “bridging hub” role within the network. Its value is reflected not only in research output, but also in its capacity to connect different regions and academic traditions. This difference can be partially explained at the institutional level. The United Kingdom has been a pioneer in community care reform since the late 1980s, and the experience accumulated through these policy initiatives has provided researchers with distinctive research topics and opportunities for comparative studies. At the policy level, the *G8 Dementia Summit* held in London in 2013 also marked the United Kingdom’s role in promoting dementia research as an international collaborative agenda [15]. At the same time, national research infrastructures such as *Join Dementia Research* have systematically reduced the organizational costs of international collaboration through a unified volunteer registration and matching system. This service enables members of the public to register their interest in dementia research and be matched with suitable studies, thereby providing an institutional mechanism for recruitment and research participation [29]. The high centrality of the United Kingdom within the network indicates that it occupies a position of structural holes [30]. This position enables it to bridge different research traditions across continental Europe, North America, and Commonwealth countries, thereby accelerating knowledge diffusion.

Australia ranked third with 142 publications and a betweenness centrality of 0.30. Its role within the network lies between that of a scale hub and a bridging hub. Australia’s relatively high betweenness centrality is associated with its distinctive geopolitical academic position. It is both a member of the Commonwealth academic community and an active participant in collaboration networks across the Asia-Pacific region, while also maintaining close personnel exchanges and project collaborations with research-intensive universities in the USA. This multiple embeddedness allows Australia to perform a cross-regional connecting function within the network.

A particularly noteworthy issue is China’s role within the network. China ranked fourth with 132 publications, yet its betweenness centrality was 0.10, lower than that of the USA and several European countries with high publication outputs. This “high-output but low-connectivity” pattern suggests that knowledge production in this field in China is mainly organized around domestic research teams, while the breadth and depth of international collaboration still leave considerable room for improvement. From a positive perspective, this suggests that China has a relatively strong capacity for autonomous knowledge production in research on cognitive impairment and well-being. From a limiting perspective, lower network connectivity may constrain the international visibility and academic influence of its research. In the section on disciplinary evolution, we observed that China has become an important emerging participant in this field in recent years. The collaboration network analysis further suggests that China is currently more of a knowledge producer than a knowledge broker, and the transformation of its network role still needs to be promoted through deliberate international collaboration strategies.

Italy also ranked among the top 10 countries in publication output, but its relatively low betweenness centrality (0.06) suggests that, within the retrieved WoSCC-indexed collaboration network, it was more visible as a productive European contributor than as a cross-regional broker. This position may reflect a more regionally embedded collaboration pattern rather than a central bridging role in the global network.

At the institutional level, the collaboration network shows a clear hierarchical structure. The University of London ranked first with 77 publications, followed by University College London with 51 publications and the University of California System with 49 publications. The University of California System ranked first among institutions in betweenness centrality, with a value of 0.14. It formed an internal collaboration network across multiple campuses, including Los Angeles, San Francisco, and San Diego, and also connected externally with research institutions in Asia and Europe. The betweenness centrality values of the University of New South Wales Sydney and Vrije Universiteit Amsterdam were both 0.08, indicating that research institutions in Australia and the Netherlands also played bridging roles in regional collaboration. In terms of the overall pattern, the institutional collaboration network was structured around large research universities in North America and Western Europe, with several Australian universities embedded as important nodes in the Southern Hemisphere. The peripheral position of Asian universities, especially Chinese universities, was also evident at the institutional level, with strong output capacity but clearly insufficient network connectivity.

Author-level collaboration network analysis revealed a core pattern of differentiation in knowledge production within this field, namely the emergence of distinct roles as “knowledge producers” and “knowledge brokers” among highly productive authors. Clare maintained a leading position on both dimensions, with 21 publications and a betweenness centrality of 0.27, combining substantial research output with strong network connectivity. Orrell, with 12 publications and a remarkably high betweenness centrality of 0.79, emerged as the most prominent bridging node in the network. His high betweenness centrality suggests that he occupied an important bridging position within the author collaboration network. Bennett also demonstrated a notable combination of productivity and connectivity, with 13 publications and a betweenness centrality of 0.47, providing further evidence for this pattern of role differentiation. In contrast, authors such as Martyr (13 publications, centrality = 0) and Subramaniam (9 publications, centrality = 0), despite their substantial research output, played almost no connecting role within the network.

Overall, several clear trends can be identified in the evolution of collaboration networks within this field. First, collaboration patterns have gradually shifted from scattered and spontaneous cooperation toward more institutionalized and platform-based forms of collaboration, with national research infrastructures and policy agendas playing an increasingly important role in shaping collaborative structures. Second, the bridging functions of core hub countries and institutions have continued to strengthen, whereas progress toward a more decentralized network structure has remained relatively slow, and emerging participant countries still occupy peripheral positions within the network. Third, the functional differentiation observed within author collaboration networks constitutes an important structural foundation for the maturation of knowledge production in this field. However, the core collaborative groups have not yet formed a fully integrated structure at the overall network level, and considerable integration potential remains to be realized. Taken together, these trends suggest that the field is currently at a critical stage of transition from an “early expansion phase” to a “deep integration phase”. How institutional innovation and resource investment are used to promote further network integration and facilitate the movement of emerging research actors from the periphery toward hub positions will directly influence the future efficiency of knowledge innovation and the academic impact of this field.

### 4.2. Research Themes and Hotspots

Within the retrieved dataset, caregiver well-being and burden constituted the largest and longest-running research theme. The close co-occurrence of cluster #0 *care professional* and cluster #1 *dementia caregiver*, the high betweenness centrality of keywords such as “family caregivers” and “burden”, and the long-term bursts of “burden” and “stress” since the mid-1990s jointly confirm the sustained prominence of this topic. Its emergence can be traced to the deinstitutionalization of mental health care in Western welfare states in the late 1980s and the policy shift toward “ageing in place”. These changes transferred the main costs of cognitive impairment care from institutions to families, especially spouses and adult daughters, thereby giving rise to a research tradition that has continued to the present day. It is worth noting that the collaboration network analysis provides new country-level evidence for this narrative: the United Kingdom showed the highest betweenness centrality in transnational collaboration (0.34), and the country was one of the pioneers in deinstitutionalization and community care reform. This suggests that a country’s policy experience influenced not only its research volume, but also the diffusion of caregiver-related issues into a global academic focus through transnational collaboration networks. In recent years, as represented by Giebel et al. [25], this research line has shifted from describing levels of burden toward constructing and validating evidence-based interventions for caregivers’ mental health. This shift echoes the transformation of cognitive impairment care from a private difficulty into a public policy issue against the background of shrinking family size and intergenerational residential separation.

The second theme focuses on the psychosocial well-being of older adults with cognitive impairment. It emerged later than caregiver research, but marks an important epistemological shift. This cluster is independent of clinical literature that investigates biomarkers and pharmacological efficacy. It is rooted in social gerontology and positive psychology, and treats concepts such as life satisfaction, well-being, and meaning in life as dependent variables. This shift is deeply aligned with Kitwood’s critique of “*malignant social psychology*” and his advocacy of “*personhood*” [31]. It grants people with cognitive impairment the status of subjects, rather than treating them merely as carriers of pathology. An early empirical example of this perspective can be traced to Clare et al.’s study of illness perceptions, coping, and well-being among people with mild cognitive impairment and their care partners [32]. That study used both patients and care partners as informants and revealed the interaction of subjective experiences within dyadic relationships. This shift did not occur in isolation. It found a corresponding structural representation in the disciplinary network: *PUBLIC, ENVIRONMENTAL and OCCUPATIONAL HEALTH* became a key bridging discipline, with a betweenness centrality of 0.35, pushing the field beyond the biomedical model toward the biopsychosocial model envisioned by Engel [33]. At the same time, micro-level individuals in the collaboration network, such as Bennett (centrality = 0.47) and Orrell (centrality = 0.79), may have played a role in knowledge integration in this process, connecting researchers oriented toward biomedical research with those oriented toward social science perspectives and accelerating the legitimization of the patient perspective. Although Ryff’s framework provides a useful theoretical reference for psychological well-being in later life, the terms captured in this study, such as quality of life, life satisfaction, mental health, positive affect, and social participation, belong to partly different conceptual traditions and should not be treated as fully interchangeable. Such semantic heterogeneity may create some risk of conceptual conflation, so the resulting knowledge map should be interpreted as a map of well-being-related research rather than as a direct representation of one unified well-being construct.

Non-pharmacological interventions have become the cluster with the most notable dynamic expansion in recent years. The burst keyword sequence clearly presents a three-stage progression: “music therapy” (2014–2018), followed by “meta-analysis” (2022–2023), and then “virtual reality” (2025–2026). These stages correspond respectively to early single-modality exploration, evidence synthesis, and the current new wave of technology-enabled interventions. The high citation frequency of the *Cochrane Database of Systematic Reviews* and the key node position of Woods et al.’s review in the co-citation network further support this judgment [24]. It is worth emphasizing that the function of systematic reviews in this field has changed. They are no longer only terminal summaries of existing evidence but have increasingly become starting points for new intervention design. New trials often directly target gaps revealed by systematic reviews, and are then incorporated into updated systematic reviews, forming an accelerated knowledge production loop. This reflexive structure of “evidence–intervention–evidence” is a key mechanism for understanding the accelerated growth of knowledge in this field. The remaining part of the map is jointly constituted by clusters related to methodological research and measurement tools, as well as clusters concerning specific populations, including Indigenous peoples, prisoners, and low- and middle-income populations. The former reflects that assessing well-being at different stages of cognitive decline remains an unresolved problem of construct validity. The latter, although limited in scale, reveals the slow growth of health equity issues at the margins of the field. One structural gap deserves particular attention. Although the global cost of cognitive impairment care has reached US$1.3 trillion, and both WHO and the Lancet Commission have repeatedly emphasized the enormous economic challenge posed by this issue [2,3], interdisciplinary research linking this field with economics remains scarce. Even within the existing body of literature, studies that explicitly treat economic well-being or care costs as core variables are only sporadically visible. For example, Hu et al. examined the gendered effects of physical disability or dementia on economic well-being and healthcare co-payments among older adults [34]. This gap is not accidental. Health economics and cognitive impairment research have long belonged to different academic communities, each with its own journals, conferences, and methodological tools. The findings of this study thus serve as a reminder: without the inclusion of an economic dimension, the knowledge structure of this field will struggle to fully respond to real-world policy needs. Future research should examine this economic dimension with a dedicated search strategy and clearer conceptual boundaries.

### 4.3. Research Trends and Future Directions

The temporal evolutionary trajectory of this field can be summarized as follows: it began with descriptive studies of caregiver burden, underwent an interventionist turn, and has moved into a recent stage characterized by technology-enabled approaches. The 2013 *G8 Dementia Summit* [15] coincided with the upward inflection point of the publication growth curve, suggesting that major policy agendas may partly shape the broader research context of academic output. The COVID-19 pandemic functioned as a major public-health shock. It intensified caregiver burden while also accelerating the application of remote and technology-mediated care models. As a result, caregiver-related clusters and technology-related clusters temporarily converged and subsequently moved toward institutionalization in the post-pandemic period. A systematic review and meta-analysis examining the psychological well-being of caregivers of people with dementia or mild cognitive impairment during the COVID-19 pandemic [35] represents a landmark product of this institutionalization process at the level of evidence synthesis. Observing recent developments in the disciplinary network helps identify future trajectories. The continuously increasing centrality of public health, together with the emergence of medical informatics as a new node, indicates that the field is simultaneously undergoing two transformations. One is an outward expansion from a biomedical-dominated paradigm toward a social-ecological framework. The other is an inward deepening from standardized intervention strategies toward data-driven personalized approaches. The tension and synergy between these two transformations will constitute an important force shaping future research agendas. The concentrated bursts of “meta-analysis” and “scoping review” in recent years further suggest that the research focus is shifting from efficacy validation toward implementation and dissemination.

At this stage, implementation science frameworks, such as the Consolidated Framework for Implementation Research (CFIR) [36] and the Theoretical Domains Framework (TDF) [37], may become key tools for bridging evidence and practice. From the perspective of collaboration structure, some highly productive authors have generated substantial research output while maintaining relatively weak network connectivity, placing them in locally concentrated collaboration zones. In contrast, bridging scholars such as Orrell and Bennett occupy positions of structural holes within the network [30]. From the perspective of small-world network theory [38], if the former group could establish intentional collaborations with the latter through inter-institutional agreements or international projects, this would not only enhance the visibility of their research but could also facilitate the emergence of breakthrough interdisciplinary outcomes. At the national level, the successful experience of the *Join Dementia Research* platform in the United Kingdom, which is deeply embedded within global collaboration networks, provides a useful reference for research communities characterized by high output but relatively low connectivity. Therefore, establishing national or even transnational research volunteer registration and matching systems may constitute an important infrastructure for improving research quality and impact.

Based on the above findings, this study proposes four directions that may be prioritized for future exploration. First, the integration of an economic analytical dimension. Future research should systematically incorporate the cost-effectiveness evaluation of non-pharmacological interventions, comparisons of long-term care financing models under different welfare regimes, and longitudinal tracking of the economic well-being of families affected by cognitive impairment, in order to fill the most prominent gap in the knowledge map. Second, the bridging of the global equity gap. This gap may also be partly amplified by WoSCC coverage, which gives greater visibility to English-language and internationally indexed journals. Given that most people with cognitive impairment live in low- and middle-income countries, while research capacity remains highly concentrated in high-income English-speaking regions, international comparative research, especially studies involving populations in Africa, Latin America, and South Asia, is critical for improving the global relevance of the field. Third, the evolution of caregiver-oriented dyadic or systems-oriented paradigms. At present, caregiver research and patient research mostly develop along parallel tracks. Future studies could more often adopt a dyadic unit of analysis, while simultaneously attending to the well-being trajectories of both parties and the bidirectional dynamics of care relationships. The large-scale IDEAL cohort study led by Clare and her team has accumulated important evidence in this direction. For example, its examination of the associations between relationship quality and life satisfaction and well-being among dementia caregiving dyads provides an empirical basis for taking dyadic relationships as intervention targets [39]. Fourth, the strengthening of methodological diversity. Although randomized controlled trials have increased markedly, cross-sectional surveys still dominate. The high centrality of qualitative methods literature in the co-citation network [26] suggests that the introduction of more longitudinal qualitative designs, experience sampling methods, and participatory action research would help capture the real patterns of fluctuation in well-being as cognitive decline progresses.

Overall, the field of cognitive impairment and well-being has developed from a relatively narrow concern with caregiving burden into a research domain that is intervention-oriented, increasingly methodologically diverse, and more deeply shaped by technological involvement. However, the absence of an economic pillar and North Atlantic centrism remain two major bottlenecks limiting the completeness of its knowledge structure and its global value. By mapping the structural landscape of this field, the present study has not only identified areas that have been sufficiently cultivated, but also delineated gaps that remain to be filled, providing a reference point for the future construction of interdisciplinary bridges.

In terms of limitations and future directions, several limitations of this study should be considered with caution. First, this study relied on WoSCC, whose sub-dataset coverage and timespans require explicit reporting and whose indexing may underestimate humanities, social science, and non-English, local, or regionally indexed scholarship, thereby potentially affecting the interpretation of findings related to China, low- and middle-income countries, and English-speaking research concentration; earlier WoSCC records may also have incomplete abstract, author-keyword, Keywords Plus, and author-address information [10,11,39,40]. The broad operationalization of well-being, with its conceptual heterogeneity, and the asymmetric TS/TI field design may also have influenced the relative visibility of different research traditions in the retrieved dataset, especially those in which cognitive impairment was the title-level focus and well-being was examined as an outcome, caregiving issue, or service-related concern. Second, the macro-level mapping provided by bibliometrics cannot replace assessments of the internal quality of individual studies. High citation frequency or high centrality does not equal high quality. The knowledge map presented in this study should be understood as an evidence-based overview, rather than a final judgment on the quality of specific studies in the field. Similarly, explanations involving policy events, collaboration roles, and database coverage should be understood as contextual interpretations rather than causal conclusions. Last, the time window of this study ended in 2026, and the strength of burst signals from 2025 to 2026 may have been influenced by recent publication cycles. Their robustness needs to be verified in subsequent research.

Future research may be deepened in the following directions. First, multiple databases such as Scopus and PubMed can be integrated for cross-validation. Second, in-depth qualitative systematic reviews or modified realist reviews can be conducted on specific hotspot clusters to reconstruct the causal mechanisms behind key evolutionary processes. Third, macro-level trend findings from bibliometric analysis can be used as a starting point, and qualitative methods, such as expert interviews and policy analysis, can be employed to explore the internal driving forces of knowledge evolution, thereby building an analytical bridge between macro-level knowledge maps and micro-level causal explanations.

## 5. Conclusions

Based on 1355 publications from the Web of Science Core Collection (WoSCC) between 1985 and 2026, this study used bibliometric methods to systematically map the knowledge structure, collaboration networks, and evolutionary trajectory of the intersection between cognitive impairment and well-being. The findings show that caregiver burden, as the earliest established research line, was closely associated with deinstitutionalization reforms in Western welfare states and has recently shifted from describing levels of burden toward constructing evidence-based intervention programs. The psychosocial well-being of patients emerged later, marking an epistemological shift from patients as objects to patients as subjects. Non-pharmacological interventions evolved through three stages: single-modality exploration, evidence synthesis, and technology-enabled development. Systematic reviews are shifting from endpoints of evidence to starting points for intervention design. Collaboration network analysis suggests that the USA and the United Kingdom occupy scale-oriented and bridging-oriented positions, while China presents a “high-output but low-connectivity” pattern. Bridging authors such as Orrell and Bennett occupy structural hole positions in the network and have promoted the integration of biomedical and social science orientations. Within the WoSCC-indexed literature, research output and collaboration networks were concentrated in high-income English-speaking regions, while low- and middle-income countries showed lower visibility; this pattern may partly reflect database coverage bias as well as uneven global research capacity [10]. The contribution of this study lies in providing a reference framework for theoretical integration and the optimization of research planning in this field.

## Figures and Tables

**Table 1:** Top 10 Publishing Journals.

Ranking	Journal	Frequency	Publisher	JIF Quartile	OA Status
1	Dementia: International Journal of Social Research and Practice	76	SAGE Publishing	Q2	Hybrid OA
2	Aging & Mental Health	48	Taylor & Francis	Q2	Hybrid OA
3	BMC Geriatrics	47	Springer Nature	Q1	Full OA
4	Journal of Alzheimer’s Disease	39	SAGE Publishing	Q2	Hybrid OA
5	The Gerontologist	35	Oxford University Press	Q1	Hybrid OA
6	International Journal of Geriatric Psychiatry	34	Wiley	Q1	Hybrid OA
7	International Psychogeriatrics	30	Cambridge University Press	Q1	Full OA
8	Journal of Clinical Nursing	23	Wiley	Q1	Hybrid OA
9	Frontiers in Psychiatry	21	Frontiers	Q2	Full OA
10	International Journal of Environmental Research and Public Health	21	MDPI	N/A	Full OA
11	Journals of Gerontology, Series B: Psychological Sciences and Social Sciences	21	Oxford University Press	Q1	Hybrid OA
12	PLOS ONE	21	Public Library of Science	Q2	Full OA
13	Frontiers in Psychology	17	Frontiers	Q1	Full OA
14	Geriatric Nursing	17	Elsevier	Q1	Hybrid OA
15	Journal of Advanced Nursing	17	Wiley	Q1	Hybrid OA

Notes: Frequency refers to the number of publications published by each journal.

**Table 2:** Top 10 Countries, Institutions, and Authors by Publication Output.

Ranking	Country	Count	BC	Organization	Count	BC	Author	Count	BC
1	USA	403	0.32	University of London	77	0.11	Linda Clare	21	0.27
2	United Kingdom	244	0.34	University College London	51	0.04	David A. Bennett	13	0.47
3	Australia	142	0.3	University of California System	49	0.14	Anthony Martyr	13	0.00
4	China	132	0.1	University of Toronto	31	0.03	Martin Orrell	12	0.79
5	Canada	111	0.05	King’s College London	31	0.04	Patricia A. Boyle	10	0.05
6	Netherlands	75	0.07	University of New South Wales Sydney	29	0.08	Catherine Quinn	10	0
7	Italy	60	0.06	University of Exeter	29	0.03	Mythily Subramaniam	9	0
8	Sweden	52	0.04	Vrije Universiteit Amsterdam	27	0.08	Edimansyah Abdin	9	0
9	Germany	49	0.08	Pennsylvania Commonwealth System of Higher Education (PCSHE)	27	0.03	Janhavi Ajit Vaingankar	9	0
10	Spain	48	0.05	State University System of Florida	27	0.05	Emma Wolverson	9	0

**Table 3:** Disciplinary Collaboration Networks.

Ranking	Category	Frequency	BC
1	GERONTOLOGY	479	0.17
2	GERIATRICS and GERONTOLOGY	429	0.17
3	PSYCHIATRY	225	0.07
4	NURSING	130	0.09
5	PUBLIC, ENVIRONMENTAL and OCCUPATIONAL HEALTH	110	0.35
6	NEUROSCIENCES	103	0.06
7	CLINICAL NEUROLOGY	99	0.07
8	HEALTH CARE SCIENCES and SERVICES	86	0.01
9	PSYCHOLOGY	76	0.06
10	MEDICINE, GENERAL and INTERNAL	64	0.08

Notes: Frequency refers to the number of publications assigned to each disciplinary field.

**Table 4:** Top 10 Cited Journals.

Ranking	Journal	Frequency
1	The Gerontologist	3151
2	Cochrane Database of Systematic Reviews	2481
3	Dementia: International Journal of Social Research and Practice	1536
4	Aging & Mental Health	1508
5	International Psychogeriatrics	1345
6	American Journal of Geriatric Psychiatry	1171
7	International Journal of Geriatric Psychiatry	1135
8	Journal of Clinical Nursing	1081
9	Journal of Alzheimer’s Disease	1070
10	Journal of the American Geriatrics Society	1029

Notes: Frequency refers to citation frequency.

**Table 5:** Top 20 Keywords.

Ranking	Keyword	Frequency	BC	Ranking	Keyword	Frequency	BC
1	older adults	461	0.07	11	care	157	0.24
2	Alzheimer’s disease	409	0.06	12	impact	109	0.26
3	people	286	0.04	13	risk	105	0.08
4	health	258	0.04	14	prevalence	100	0.00
5	quality of life	256	0	15	family caregivers	93	0.40
6	dementia	236	0.08	16	burden	89	0.57
7	older people	175	0.05	17	intervention	88	0.24
8	cognitive impairment	165	0.3	18	physical activity	82	0.03
9	depression	162	0.16	19	scale	80	0.26
10	mild cognitive impairment	160	0.08	20	mental health	80	0.11

Notes: Frequency refers to keyword occurrence frequency.

## Data Availability

The data presented in this study were retrieved from the Web of Science Core Collection database. The bibliographic data used for the analyses are available from the corresponding author upon reasonable request.

## Supplementary Materials

The following supporting information can be downloaded at: https://www.mdpi.com/article/10.3390/healthcare14152243/s1, Figure S1. Study Selection Flow Diagram. Table S1. Representative documents of the 15 document co-citation clusters [41,42,43,44,45,46,47,48,49,50,51,52].

## Abbreviations

The following abbreviations are used in this manuscript:

ADI	Alzheimer’s Disease International
BC	Betweenness Centrality
GBD	Global Burden of Disease
MCI	Mild Cognitive Impairment
USA	United States of America
WHO	World Health Organization
WoSCC	Web of Science Core Collection

## References

[1] Alzheimer’s Disease International (2024). World Alzheimer Report 2024: Global Changes in Attitudes to Dementia.

[2] World Health Organization (2021). Global Status Report on the Public Health Response to Dementia.

[3] Livingston G., Huntley J., Sommerlad A., Ames D., Ballard C., Banerjee S., Brayne C., Burns A., Cohen-Mansfield J., Cooper C. (2020). Dementia prevention, intervention, and care: 2020 report of the Lancet Commission. Lancet.

[4] Jennings L.A., Palimaru A., Corona M.G., Cagigas X.E., Ramirez K.D., Zhao T., Hays R.D., Wenger N.S., Reuben D.B. (2017). Patient and caregiver goals for dementia care. Qual. Life Res..

[5] Ryff C.D. (1995). Psychological well-being in adult life. Curr. Dir. Psychol. Sci..

[6] Elias L., Saroufine K., Alagha M., Beaini C., Albekai E., Semaan S., Elkhoury E., Harb F., Azar S., El Khoury N.B. (2026). The psychiatric-metabolic interface: Exploring the interplay between mental disorders and metabolic syndrome. Ment. Illn..

[7] Yang M., Kong D., Huang J., Huang Y., Sun Y., Li Z. (2025). Meta-analysis of brain structure in magnetic resonance imaging studies of adolescents with schizophrenia. Ment. Illn..

[8] Zhu J., Liu W. (2020). A tale of two databases: The use of Web of Science and Scopus in academic papers. Scientometrics.

[9] Li K., Rollins J., Yan E. (2018). Web of Science use in published research and review papers 1997–2017: A selective, dynamic, cross-domain, content-based analysis. Scientometrics.

[10] Mongeon P., Paul-Hus A. (2016). The journal coverage of Web of Science and Scopus: A comparative analysis. Scientometrics.

[11] Liu W. (2019). The data source of this study is Web of Science Core Collection? Not enough. Scientometrics.

[12] Chen C. (2006). CiteSpace II: Detecting and visualizing emerging trends and transient patterns in scientific literature. J. Am. Soc. Inf. Sci. Technol..

[13] van Eck N.J., Waltman L. (2010). Software survey: VOSviewer, a computer program for bibliometric mapping. Scientometrics.

[14] Liu W. (2021). Caveats for the use of Web of Science Core Collection in old literature retrieval and historical bibliometric analysis. Technol. Forecast. Soc. Change.

[15] G8 Health Ministers (2013). G8 Dementia Summit Declaration.

[16] De Pue S., Gillebert C., Dierckx E., Vanderhasselt M.A., De Raedt R., Van den Bussche E. (2021). The impact of the COVID-19 pandemic on wellbeing and cognitive functioning of older adults. Sci. Rep..

[17] Moreno-Agostino D., Di Gessa G. (2026). Mental health in adults aged 50+ since the COVID-19 pandemic: Are we (all) back to ‘normal’? Evidence from England. J. Affect. Disord. Rep..

[18] Yi L.R.S., Su J.J. (2023). Effects of mindfulness-based interventions on neuropsychiatric symptoms and psychological well-being in people with subjective cognitive decline and mild cognitive impairment: A meta-analysis. Int. J. Geriatr. Psychiatry.

[19] Nichols E., Steinmetz J.D., Vollset S.E., Fukutaki K., Chalek J., Abd-Allah F., Abdoli A., Abualhasan A., Abu-Gharbieh E., Akram T.T. (2022). Estimation of the global prevalence of dementia in 2019 and forecasted prevalence in 2050: An analysis for the Global Burden of Disease Study 2019. Lancet Public Health.

[20] Alzheimer’s Association (2023). 2023 Alzheimer’s disease facts and figures. Alzheimer’s Dement..

[21] World Health Organization, Alzheimer’s Disease International (2012). Dementia: A Public Health Priority.

[22] Amariglio R.E., Becker J.A., Carmasin J., Wadsworth L.P., Lorius N., Sullivan C., Maye J.E., Gidicsin C., Pepin L.C., Sperling R.A. (2012). Subjective cognitive complaints and amyloid burden in cognitively normal older individuals. Neuropsychologia.

[23] Särkämö T., Tervaniemi M., Laitinen S., Numminen A., Kurki M., Johnson J.K., Rantanen P. (2014). Cognitive, emotional, and social benefits of regular musical activities in early dementia: Randomized controlled study. Gerontologist.

[24] Woods B., O’Philbin L., Farrell E.M., Spector A.E., Orrell M. (2018). Reminiscence therapy for dementia. Cochrane Database Syst. Rev..

[25] Giebel C., Lord K., Cooper C., Shenton J., Cannon J., Pulford D., Shaw L., Gaughan A., Tetlow H., Butchard S. (2021). A UK survey of COVID-19 related social support closures and their effects on older people, people with dementia, and carers. Int. J. Geriatr. Psychiatry.

[26] Miles M.B., Huberman A.M., Saldaña J. (2014). Qualitative Data Analysis: A Methods Sourcebook.

[27] Camic P.M., Tischler V., Pearman C.H. (2014). Viewing and making art together: A multi-session art-gallery-based intervention for people with dementia and their carers. Aging Ment. Health.

[28] Camic P.M., Williams C.M., Meeten F. (2013). Does a ‘Singing Together Group’ improve the quality of life of people with a dementia and their carers? A pilot evaluation study. Dementia.

[29] Kotting P., Smith A., O’Hare M.B., Giebel C., Mendis L., Shaw C., Shillito I., Rossor M.N. (2021). A national open-access research registry to improve recruitment to clinical studies. Alzheimer’s Dement..

[30] Burt R.S. (1992). Structural Holes: The Social Structure of Competition.

[31] Kitwood T. (1997). Dementia Reconsidered: The Person Comes First.

[32] Clare L., Rowlands J., Bruce E., Surr C., Downs M. (2008). Perceptions of illness, coping, and well-being in persons with mild cog-nitive impairment and their care partners. Alzheimer Dis. Assoc. Disord..

[33] Engel G.L. (1977). The need for a new medical model: A challenge for biomedicine. Science.

[34] Hu Y., Carr P.R., Liew D., Broder J., Callander E.J., McNeil J.J. (2022). How does the onset of physical disability or dementia in older adults affect economic wellbeing and co-payments for health care? The impact of gender. BMC Geriatr..

[35] Soysal P., Veronese N., Smith L., Chen Y., Akpinar Soylemez B., Coin A., Religa D., Välimäki T., Alves M., Shenkin S.D. (2023). The impact of the COVID-19 pandemic on the psychological well-being of caregivers of people with dementia or mild cognitive impairment: A systematic review and meta-analysis. Geriatrics.

[36] Damschroder L.J., Aron D.C., Keith R.E., Kirsh S.R., Alexander J.A., Lowery J.C. (2009). Fostering implementation of health ser-vices research findings into practice: A consolidated framework for advancing implementation science. Implement. Sci..

[37] Cane J., O’Connor D., Michie S. (2012). Validation of the theoretical domains framework for use in behaviour change and imple-mentation research. Implement. Sci..

[38] Newman M.E.J. (2001). The structure of scientific collaboration networks. Proc. Natl. Acad. Sci. USA.

[39] Rippon I., Quinn C., Martyr A., Morris R., Nelis S.M., Jones I.R., Victor C.R., Clare L., on behalf of the IDEAL Programme (2020). The impact of relationship quality on life satisfaction and well-being in dementia caregiving dyads: Findings from the IDEAL study. Aging Ment. Health.

[40] Liu W., Hu G., Tang L. (2018). Missing author address information in Web of Science: An explorative study. J. Inf..

[41] Kuiper J.S., Zuidersma M., Oude Voshaar R.C., Zuidema S.U., van den Heuvel E.R., Stolk R.P., Smidt N. (2015). Social relationships and risk of dementia: A systematic review and meta-analysis of longitudinal cohort studies. Ageing Res. Rev..

[42] Wisniewski S.R., Belle S.H., Coon D.W., Marcus S.M., Ory M.G., Burgio L.D., Burns R., Schulz R., REACH Investigators (2003). The Resources for Enhancing Alzheimer’s Caregiver Health (REACH): Project design and baseline characteristics. Psychol. Aging.

[43] Olazarán J., Reisberg B., Clare L., Cruz I., Peña-Casanova J., del Ser T., Woods B., Beck C., Auer S., Lai C. (2010). Nonpharmacological therapies in Alzheimer’s disease: A systematic review of efficacy. Dement. Geriatr. Cogn. Disord..

[44] Verbeek H., Zwakhalen S.M.G., van Rossum E., Ambergen T., Kempen G.I.J.M., Hamers J.P.H. (2010). Small-scale, homelike facilities versus regular psychogeriatric nursing home wards: A cross-sectional study into residents’ characteristics. BMC Health Serv. Res..

[45] Woods R.T., Nelis S.M., Martyr A., Roberts J., Whitaker C.J., Markova I., Roth I., Morris R., Clare L. (2014). What contributes to a good quality of life in early dementia? Awareness and the QoL-AD: A cross-sectional study. Health Qual. Life Outcomes.

[46] Beerens H.C., Sutcliffe C., Renom-Guiteras A., Soto M.E., Suhonen R., Zabalegui A., Bökberg C., Saks K., Hamers J.P.H. (2014). Quality of life and quality of care for people with dementia receiving long-term institutional care or professional home care: The European RightTimePlaceCare Study. J. Am. Med. Dir. Assoc..

[47] Mordoch E., Osterreicher A., Guse L., Roger K., Thompson G. (2013). Use of social commitment robots in the care of elderly people with dementia: A literature review. Maturitas.

[48] Wilson R.S., Krueger K.R., Arnold S.E., Schneider J.A., Kelly J.F., Barnes L.L., Tang Y., Bennett D.A. (2007). Loneliness and risk of Alzheimer disease. Arch. Gen. Psychiatry.

[49] Baumgarten M., Battista R.N., Infante-Rivard C., Hanley J.A., Becker R., Gauthier S. (1992). The psychological and physical health of family members caring for an elderly person with dementia. J. Clin. Epidemiol..

[50] Prince M., Bryce R., Albanese E., Wimo A., Ribeiro W., Ferri C.P. (2013). The global prevalence of dementia: A systematic review and metaanalysis. Alzheimer’s Dement..

[51] Fuller-Jonap F., Haley W.E. (1995). Mental and physical health of male caregivers of a spouse with Alzheimer’s disease. J. Aging Health.

[52] Chan D., Livingston G., Jones L., Sampson E.L. (2013). Grief reactions in dementia carers: A systematic review. Int. J. Geriatr. Psychiatry.

