# Supplementary material for: The Evolution of Cognitive Impairment and Wellbeing Research: A Bibliometric Analysis
Source: Healthcare (Basel). 2026 Jul 23;14(15):2243. doi: 10.3390/healthcare14152243 (PMC13463958; doi:10.3390/healthcare14152243)
Supplement: Supplementary file 1 [file healthcare-14-02243-s001.zip › healthcare-4409198-supplementary.pdf]

Supplementary File

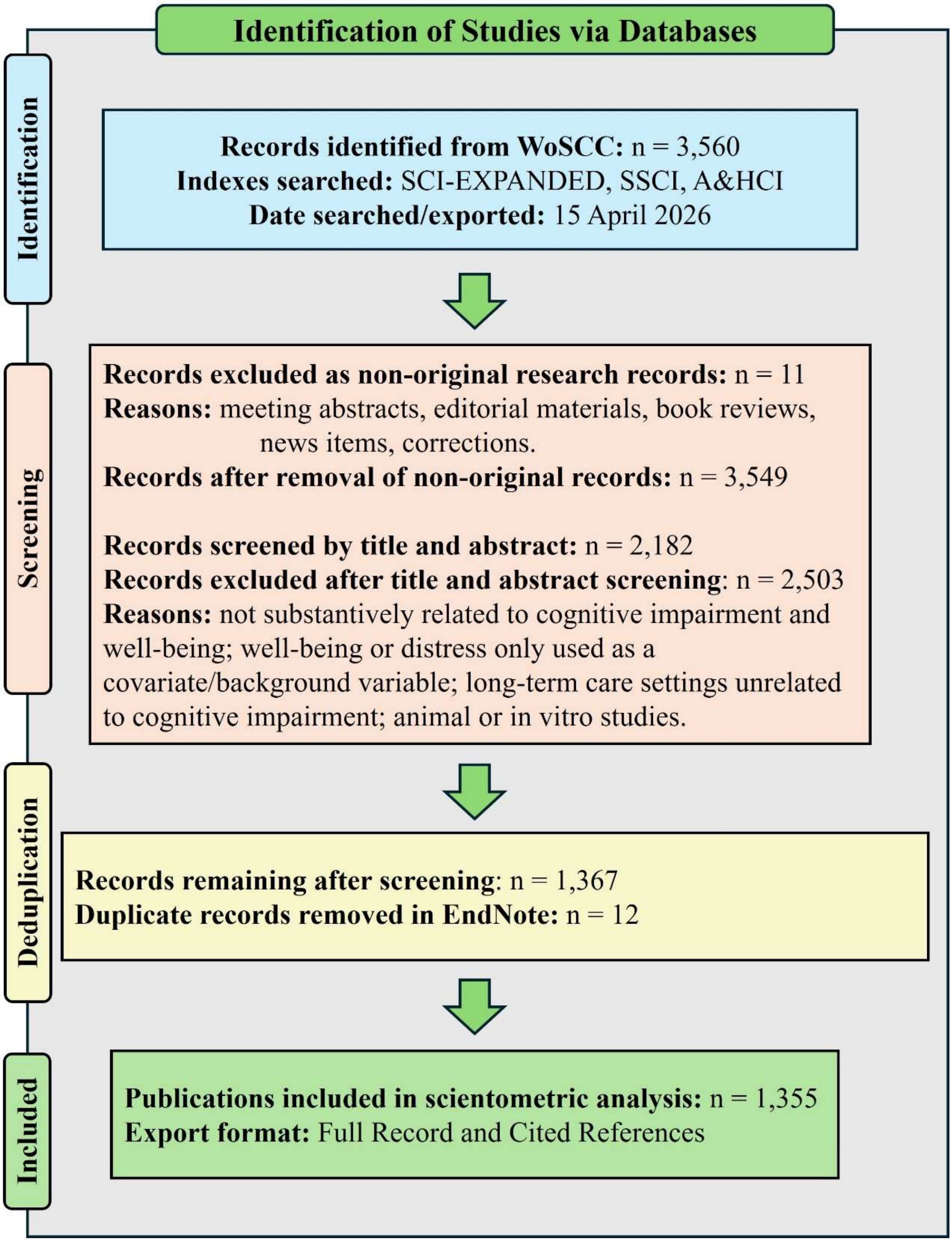

Figure S1. Study Selection Flow Diagram

Note. The flow diagram reports the literature identification, screening, deduplication, and final inclusion process for the scientometric analysis. The diagram was prepared with reference to the PRISMA 2020 flow diagram for transparent reporting of record selection.

Table S1. Representative documents of the 15 document co-citation clusters

Cluster	Representative Document	Year	Frequency	BC
#0 dementia	Livingston G, 2020, LANCET, V396, P413, DOI 10.1016/S0140-6736(20)30367-6	74	0	2020
#1 mere pleasure	Kuiper JS, 2015, AGEING RES REV, V22, P39, DOI 10.1016/j.arr.2015.04.006	8	0.08	2015
#2 mental health effects intervention strategies	Wisniewski SR, 2003, PSYCHOL AGING, V18, P375, DOI 10.1037/0882-7974.18.3.375	5	0.09	2003
#3 action plan	Olazarán J, 2010, DEMENT GERIATR COGN, V30, P161, DOI 10.1159/000316119	6	0.07	2010
#4 stress mood sleep	Särkämö T, 2014, GERONTOLOGIST, V54, P634, DOI 10.1093/geront/gnt100	10	0.61	2014
#5 nursing home resident	Verbeek H, 2010, BMC HEALTH SERV RES, V10, P0, DOI 10.1186/1472-6963-10-30	6	0.03	2010
#6 enhancing active life	Woods RT, 2014, HEALTH QUAL LIFE OUT, V12, P0, DOI 10.1186/1477-7525-12-94	4	0.06	2014
#7 depression anxiety	Beerens HC, 2014, J AM MED DIR ASSOC, V15, P54, DOI 10.1016/j.jamda.2013.09.010	4	0.02	2014
#8 high-intensity exercise program	Mordoch E, 2013, MATURITAS, V74, P14, DOI 10.1016/j.maturitas.2012.10.015	4	0.02	2013
#9 motor function	Wilson RS, 2007, ARCH GEN PSYCHIAT, V64, P234, DOI 10.1001/archpsyc.64.2.234	3	0	2007
#10 prevalence correlate	BAUMGARTEN M, 1992, J CLIN EPIDEMIOL, V45, P61, DOI 10.1016/0895-4356(92)90189-T	3	0.05	1992
#11 caregiver health	Prince M, 2013, ALZHEIMERS DEMENT, V9, P63, DOI 10.1016/j.jalz.2012.11.007	17	0	2013
#12 late-life dementia	FULLERJONAP F, 1995, J AGING HEALTH, V7, P99, DOI 10.1177/089826439500700105	3	0.06	1995
#13 caring relationship	Chan D, 2013, INT J GERIATR PSYCH, V28, P1, DOI 10.1002/gps.3795	3	0.08	2013
#14 subjective need	Miles MB, 2014, QUALITATIVE DATA ANALYSIS: AN EXPANDED SOURCEBOOK, VThird, P0	4	0.89	2014

Note: The representative document refers to the most frequently cited member within each co-citation cluster. Citation frequency and BC were derived from the CiteSpace document co-citation analysis.
